# STING1 Promotes Ferroptosis Through MFN1/2-Dependent Mitochondrial Fusion

**DOI:** 10.3389/fcell.2021.698679

**Published:** 2021-06-14

**Authors:** Changfeng Li, Jiao Liu, Wen Hou, Rui Kang, Daolin Tang

**Affiliations:** ^1^Department of Endoscopy Center, China-Japan Union Hospital of Jilin University, Changchun, China; ^2^The Third Affiliated Hospital, Guangzhou Medical University, Guangzhou, China; ^3^Department of Radiation Oncology, University of Pittsburgh, Pittsburgh, PA, United States; ^4^Department of Surgery, UT Southwestern Medical Center, Dallas, TX, United States

**Keywords:** MFN1/2, ferroptosis, mitochondria, dynamic, STING1

## Abstract

Ferroptosis is a type of iron-dependent regulated cell death caused by the disruption that occurs when oxidative stress and antioxidant defenses interact, and then driven by lipid peroxidation and subsequent plasma membrane ruptures. The regulation of ferroptosis involves many factors, including the crosstalk between subcellular organelles, such as mitochondria, endoplasmic reticulum (ER), lysosomes, lipid droplets, and peroxisomes. Here, we show that the ER protein STING1 (also known as STING or TMEM173) promotes ferroptosis in human pancreatic cancer cell lines by increasing MFN1/2-dependent mitochondrial fusion, but not mitophagy-mediated mitochondrial removal. The classic ferroptosis inducer erastin, but not sulfasalazine, induces the accumulation of STING1 in the mitochondria, where it binds to MFN1/2 to trigger mitochondrial fusion, leading to subsequent reactive oxygen species production and lipid peroxidation. Consequently, *in vitro* or xenograft mouse models show that the genetic depletion of STING1 or MFN1/2 (but not the mitophagy regulator PINK1 or PRKN) reduces the sensitivity of pancreatic cancer cells to ferroptosis. These findings not only establish a new mitochondrial fusion-dependent cell death mechanism, but also indicate a potential strategy for enhancing ferroptosis-based therapy.

## Introduction

Mitochondria are dynamic membrane-bound organelles that are not only the main site of energy metabolism, but also participate in various cell signal transduction processes by regulating oxidative stress and calcium homeostasis (Friedman and Nunnari, 2014). Under normal conditions, mitochondria are altered through balanced fission and fusion. Once this dynamic balance is broken, the mitochondria may show functional changes, and even defects (Giacomello et al., 2020). The state of mitochondrial fission and fusion is closely related to cell survival or death, which is further regulated by mitochondrial quality control systems (Bock and Tait, 2020). For example, mitophagy is a selective autophagic degradation pathway that removes damaged mitochondria when the level of fragmented mitochondria increases (Palikaras et al., 2018). The destruction of mitochondrial dynamic regulation can lead to a variety of diseases, including cancer (Giacomello et al., 2020). Understanding the mechanism and regulation of mitochondrial dynamics may lead to the development of new cancer treatment strategies.

Stimulator of interferon response CGAMP interactor 1 (STING1, also known as STING or TMEM173) is an endoplasmic reticulum (ER) protein that promotes innate immune signal transduction in response to host DNA damage and pathogen invasion (Motwani et al., 2019). In addition to immune function, STING1 also regulates various types of cell death under different experimental conditions, including apoptosis, pyroptosis, necroptosis, mitosis, and ferroptosis (Murthy et al., 2020). Ferroptosis is an iron-dependent non-apoptotic cell death (Dixon et al., 2012) caused by the imbalance between oxidative stress and antioxidant systems, and has complex molecular mechanisms and biochemical cascades (Chen et al., 2020). Many classic ferroptosis activators are inhibitors of endogenous antioxidant systems, especially the system xc^–^-glutathione peroxidase 4 (GPX4) pathway (Dixon et al., 2012; Yang et al., 2014). Non-classical activators of ferroptosis include physiological or pathological stresses, such as heat (Distefano et al., 2017), hypoxia (An et al., 2020), circadian rhythm disturbances (Liu Y. et al., 2020), and DNA damage (Song et al., 2016; Lei et al., 2020). We previously demonstrated that STING1 is a promoter of ferroptotic cancer cell death caused by zalcitabine-induced mitochondrial DNA damage (Li C. et al., 2020). However, there is no research on whether STING1 can regulate mitochondrial dynamics to control ferroptosis.

In the study described here, we provide the first evidence that STING1 promotes erastin-induced ferroptosis in human pancreatic cancer cell lines by promoting mitochondrial fusion via binding to mitofusins (including mitofusin 1 [MFN1] and mitofusin 2 [MFN2]), which are key regulators of mitochondrial dynamics. In contrast, classic PTEN-induced kinase 1 (PINK1)-dependent mitophagy is not essential for erastin-induced ferroptosis. Consequently, the genetic inactivation of the STING1-dependent mitochondrial fusion pathway limits anticancer activity of ferroptosis activators *in vitro* and *in vivo*. Together, these findings demonstrate a new function of STING1 in modulating mitochondrial dynamics and cell death.

## Results

### Erastin Induces Mitochondrial Translocation of STING1 During Ferroptosis

**FIGURE 1:**
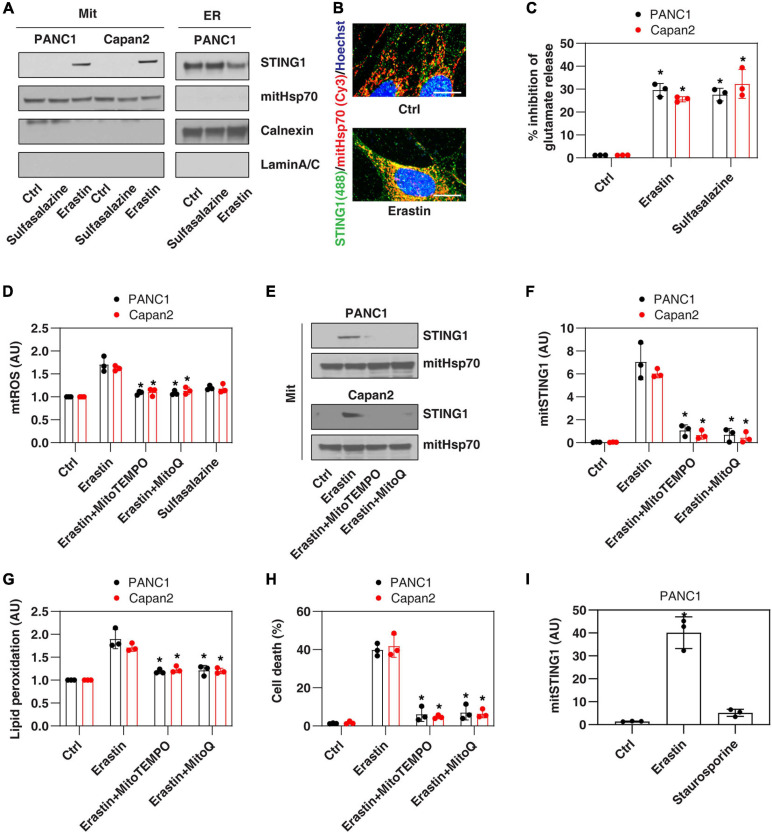
Erastin induces mitochondrial translocation of STING1 during ferroptosis. **(A)** Western blot analysis of STING1 expression in isolated mitochondria or ER in indicated cells following erastin (10 μM) or sulfasalazine (500 μM) treatment for 24 h. Antibodies to mitHsp70, calnexin and laminA/C were used as markers for mitochondria, ER and nucleus, respectively. **(B)** Immunofluorescence analysis of mitochondrial localization of STING1 in PANC1 cells in response to erastin (10 μM) for 24 h (Green: STING1; red: mitHsp70; blue: hoechst 33258; bar = 20 μm). **(C)** Analysis of glutamate release from PANC1 or Capan2 cells in response to erastin (10 μM) or sulfasalazine (500 μM) for 24 h (*n* = 3 wells/group; ANOVA with Tukey’s multiple comparisons test; *P < 0.05 versus control group). **(D–H)** PANC1 or Capan2 cells were treated with erastin (10 μM) in the absence or presence of MitoTEMPO (100 μM) or MitoQ (10 μM) for 24 h. The levels of mtROS, mitochondrial STING1, lipid peroxidation, and cell death were assayed (*n* = 3 wells/group; ANOVA with Tukey’s multiple comparisons test; *P < 0.05 versus erastin group). **(I)** Analysis of the level of mitochondrial STING1 in PANC1 cells in response to erastin (10 μM) or staurosporine (200 nM) for 24 h (*n* = 3 wells/group; ANOVA with Tukey’s multiple comparisons test; *P < 0.05 versus control group). Data are from two or three independent experiments.

Since the ER membrane and mitochondrial membrane are in close contact during cell stresses (Bravo-Sagua et al., 2013), we first investigated whether the ER protein STING1 can be translocated to mitochondria in PANC1, a human pancreatic ductal adenocarcinoma (PDAC) cell line that expresses STING1 and is sensitive to ferroptosis (Zhu et al., 2017; Li C. et al., 2020). We used a classic ferroptosis activator, erastin, which not only binds to the amino acid antiporter system xc^–^ in the plasma membrane to inhibit cystine uptake with a 1:1 counter-transport of glutamate (Dixon et al., 2012), but also binds to voltage-dependent anion channels (VDACs) on the mitochondria (Yagoda et al., 2007), thereby increasing the permeability of the mitochondrial membrane and oxidative damage. Western blot analysis observed that after erastin treatment, the accumulation of STING1 in mitochondria increased, whereas STING1 expression in ER decreased (Figure 1A). Consistently, immunofluorescence analysis showed that erastin increased the localization of STING1 in the mitochondria of PANC1 cells (Figure 1B).

To define the mechanism of STING1 mitochondrial translocation, we treated PANC1 cells with sulfasalazine, which is a classic system xc^–^ inhibitor with activation that induces ferroptosis (Dixon et al., 2012). Unlike erastin, sulfasalazine failed to induce mitochondrial translocation of STING1 (Figure 1A), indicating that inhibiting system xc^–^ is not essential for mitochondrial translocation of STING1 during ferroptosis. Similarly, erastin (but not sulfasalazine) induced STING1 mitochondrial translocation in another human PDAC cell line (Capan2) (Figure 1A). As a control, we measured the inhibition of glutamate release and found that erastin and sulfasalazine both inhibited system xc^–^ activity in PANC1 and Capan2 cells (Figure 1C).

Since erastin can cause mitochondrial damage (Dixon et al., 2012), we further explored whether erastin-induced STING1 mitochondrial translocation requires a mitochondrial reactive oxygen species (mtROS) signal. To test this hypothesis, we used MitoTEMPO and MitoQ, which are mitochondria-targeted superoxide dismutase mimetic with superoxide and alkyl radical scavenging properties (Liang et al., 2010). MitoTEMPO and MitoQ inhibited erastin-induced mtROS production (Figure 1D), the accumulation of STING1 in the mitochondria (Figures 1E,F), lipid peroxidation (Figure 1G), and cell death (Figure 1H). It is worth noting that staurosporine, a classic apoptosis inducer, failed to induce STING1 mitochondrial translocation (Figure 1I). Collectively, these findings indicate that erastin induces mitochondrial translocation of STING1 by activating mitochondrial oxidative stress.

### STING1 Promotes Erastin-Induced Ferroptosis With Increased Mitochondrial Fusion

**FIGURE 2:**
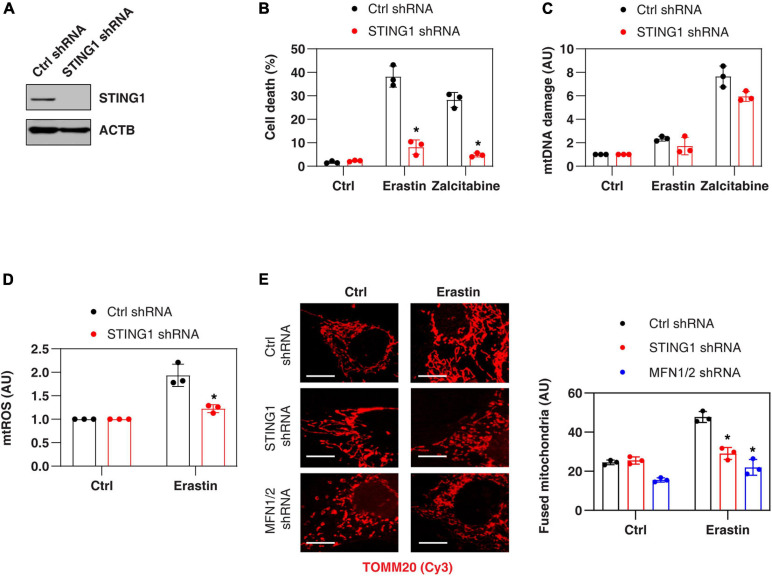
STING1 promotes erastin-induced ferroptosis with increased mitochondrial fusion. **(A)** Western blot analysis of STING1 expression in control and STING^*KD*^ PANC1 cells. **(B–E)** Indicated PANC1 cells were treated with erastin (10 μM, 24 h) or zalcitabine (20 μM, 72 h), and cell death, mitochondrial DNA damage, mtROS, and mitochondrial morphology were assayed (*n* = 3 wells/group; ANOVA with Tukey’s multiple comparisons test; *P < 0.05 versus control shRNA group; bar = 20 μm). Data are from two or three independent experiments.

Consistent with previous findings that STING1 is a positive regulator of zalcitabine-induced ferroptosis (Li C. et al., 2020), the knockdown of STING1 by two different shRNA (termed as STING1^*KD*^) (Figure 2A and Supplementary Figure 1) also limited erastin-induced cell death in PANC1 cells (Figure 2B and Supplementary Figure 1). Zalcitabine acts as a nucleoside deoxycytidine analog to induce ferroptosis by activating mitochondrial DNA damage (Li C. et al., 2020). However, erastin failed to cause significant mitochondrial DNA damage (Figure 2C) but did increase the production of mtROS (Figure 2D). Moreover, the knockdown of STING1 failed to affect zalcitabine-induced mitochondrial DNA damage (Figure 2C). These findings indicate that STING1-mediated erastin-induced ferroptosis may not be dependent on the mitochondrial DNA damage response. Moreover, STING1 failed to affect sulfasalazine-induced cell death (Supplementary Figure 1).

To define the mechanism of action of STING1 in ferroptotic cell death, we further analyzed the mtROS production and mitochondrial morphology (by staining with translocase of outer mitochondrial membrane 20 [TOMM20/TOM20]) of mitochondria in control and STING^*KD*^ cells. Erastin-induced mtROS production was inhibited in STING1^*KD*^ cells (Figure 2D), indicating that STING1 regulates mtROS production during ferroptosis. Unlike mitochondrial fission, which is one of morphological events during apoptotic cell death (Youle and Karbowski, 2005), mitochondrial fusion was significantly increased in erastin-treated control cells, but not STING^*KD*^ PANC1 cells (Figure 2E). These analyses indicate a potential role of STING1-dependent mitochondrial fusion in promoting ferroptosis.

### STING Binds MFN1/2 to Promote Ferroptosis Through Mitochondria Fusion

**FIGURE 3:**
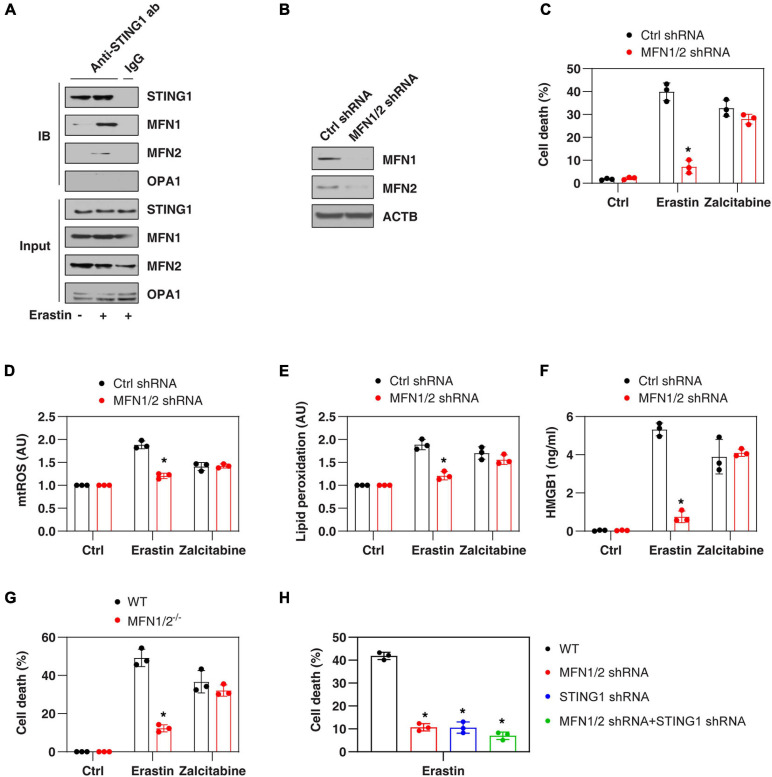
MFN1/2 promotes erastin-induced ferroptosis. **(A)** Immunoprecipitation assay of the interaction between STING1, MFN1, MFN2, and OPA1 in mitochondria fractions of PANC1 cells with or without erastin (10 μM) treatment for 12 h. **(B)** Western blot analysis of MFN1 and MFN2 expression in control and MFN1/2^*KD*^ PANC1 cells. **(C–F)** Indicated PANC1 cells were treated with erastin (10 μM, 24 h) or zalcitabine (20 μM, 72 h), and then cell death, intracellular mtROS, intracellular lipid peroxidation, and extracellular HMGB1 were assayed (*n* = 3 wells/group; ANOVA with Tukey’s multiple comparisons test; *P < 0.05 versus control shRNA group). **(G)** Indicated MEFs cells were treated with erastin (5 μM, 24 h) or zalcitabine (20 μM, 72 h) and then cell death was assayed (*n* = 3 wells/group; ANOVA with Tukey’s multiple comparisons test; *P < 0.05 versus WT group). **(H)** Indicated PANC1 cells were treated with erastin (10 μM) for 24 h and then cell death was assayed (*n* = 3 wells/group; ANOVA with Tukey’s multiple comparisons test; *P < 0.05 versus control shRNA group). Data are from two or three independent experiments.

Mitochondrial fusion requires the coordinated fusion of outer and inner membranes, which is mainly driven by OPA1 mitochondrial dynamin-like GTPase (OPA1) and mitofusins (including MFN1 and MFN2), respectively (Giacomello et al., 2020). Unexpectedly, an immunoprecipitation assay showed a direct interaction between STING1, MFN1, and MFN2 in the mitochondrial fraction of PANC1 cells following erastin treatment (Figure 3A). In contrast, erastin failed to cause an interaction between STING1 and OPA1 (Figure 3A). As expected, the knockdown of MFN1 and MFN2 (hereafter MFN1/2^*KD*^) also limited erastin-induced mitochondrial fusion in PANC1 cells (Figure 2E). These findings suggest that STING1 is a regulator of mitofusin-mediated mitochondrial fusion machinery in ferroptosis.

We next investigated whether mitofusins have a function like STING1 in the regulation of ferroptotic response. Indeed, the suppression of MFN1/2 by shRNAs (Figure 3B) also inhibited erastin-induced cell death (Figure 3C) in PANC1 cells with reduced mtROS production (Figure 3D), decreased lipid peroxidation (Figure 3E), and diminished release of high mobility group box 1 (HMGB1, a representative damage-associated molecular pattern during ferroptotic cell death (Wen et al., 2019; Figure 3F). Compared with the control group, MFN1/2^*KD*^ PANC1 cells had similar sensitivity to zalcitabine-induced ferroptotic response (Figures 3C–F), indicating that the mitochondrial fusion machinery may not be involved in the regulation of mitochondrial DNA damage-induced ferroptosis. Moreover, erastin-induced cell death was also blocked in MFN1/2^–/–^ mouse embryonic fibroblasts (MEFs) (Figure 3G). Compared with the knockdown of MFN1/2 or STING1 alone, double knockdown of STING1 and MFN1/2 had similar inhibitory effects on erastin-induced cell death (Figure 3H). Together, these findings support the idea that a mitochondrial STING1-MFN1/2 complex promotes ferroptosis by mitochondria fusion-mediated mtROS production and lipid peroxidation.

### PINK1-Dependent Mitophagy Is Not Involved in Regulating Erastin-Induced Ferroptosis

**FIGURE 4:**
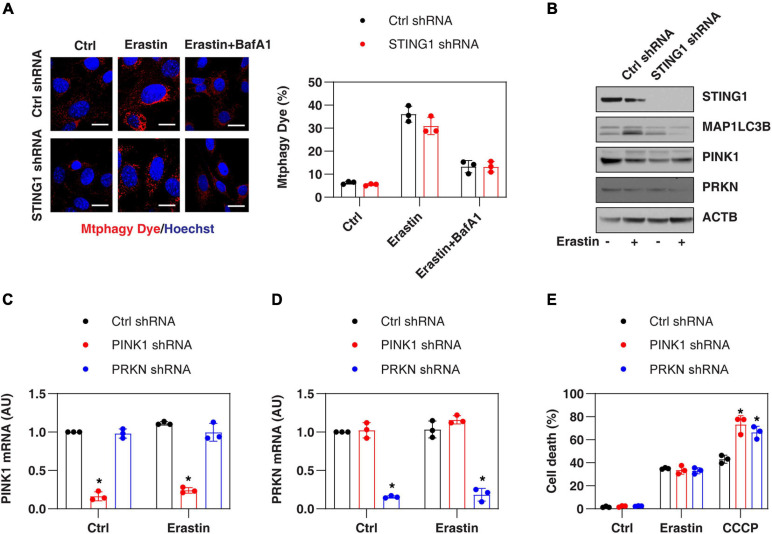
PINK1-dependent mitophagy is not involved in regulating erastin-induced ferroptosis. **(A,B)** Indicated control and STING^*KD*^ PANC1 cells were treated with erastin (10 μM, 24 h) with or without bafilomycin A1 (BafA1, 50 nM) and then mitophagy (stained with Mtphagy Dye) and protein expression were assayed (*n* = 3 wells/group; ANOVA with Tukey’s multiple comparisons test; bar = 20 μm). **(C,D)** qPCR analysis of PINK1 and PRKN expression in control, PINK1^*KD*^, or PRKN^*KD*^ PANC1 cells with or without erastin (10 μM, 24 h) treatment (*n* = 3 wells/group; ANOVA with Tukey’s multiple comparisons test; *P < 0.05 versus control shRNA group). **(E)** Indicated PANC1 cells were treated with erastin (10 μM) or CCCP (10 μM) for 24 h and cell death was assayed (*n* = 3 wells/group; ANOVA with Tukey’s multiple comparisons test; *P < 0.05 versus control shRNA group). Data are from two or three independent experiments.

Since STING1 is a promoter of autophagosome formation (Gui et al., 2019), and mitophagy contributes to the clearing of damaged mitochondria (Palikaras et al., 2018), we investigated whether STING1 is involved in ferroptosis by regulating mitophagy. Mitophagy was assayed by a commercial small molecule fluorescent probe, Mtphagy Dye, which was recently developed by Dojindo Laboratories (Iwashita et al., 2017). Under normal circumstances, Mtphagy Dye accumulated in the mitochondria and its fluorescence was weak (Iwashita et al., 2017; Figure 4A). When mitophagy was induced by erastin, the damaged mitochondria fused to lysosomes, causing the Mtphagy Dye to emit a high fluorescent signal (Iwashita et al., 2017; Figure 4A). This process was inhibited by bafilomycin A1 (BafA1) (Figure 4A), which is a vacuolar H + ATPase inhibitor used to block autophagic flux by decreasing autophagosome-lysosome fusion (Li J. et al., 2020). Although STING1^*KD*^ cells had lower microtubule-associated protein 1 light chain 3 beta-II (MAP1LC3B-II, a marker of autophagosome formation) expression (Figure 4B), there was no significant difference in the upregulation of Mtphagy Dye induced by erastin compared with control cells (Figure 4A).

PINK1 is a key regulator of mitophagy in mammalian cells by parkin RBR E3 ubiquitin protein ligase (PRKN, also known as PARK2) (Geisler et al., 2010). Erastin and STING1 depletion failed to affect the expression of PINK1 and PRKN in PANC1 cells (Figure 4B). In addition, the knockdown of PINK1 or PRKN by shRNA (Figures 4C,D) promoted uncoupler carbonyl cyanide m-chlorophenylhydrazone (CCCP, a classical mitophagy inducer)-induced cell death, but failed to affect erastin-induced cell death (Figure 4E). Collectively, these findings suggest that the classical PINK1-dependent mitophagy pathway is not involved in regulating erastin-induced ferroptosis.

### Inhibiting the STING1-MFN1/2 Pathway Limits Ferroptosis Therapy *in vivo*

**FIGURE 5:**
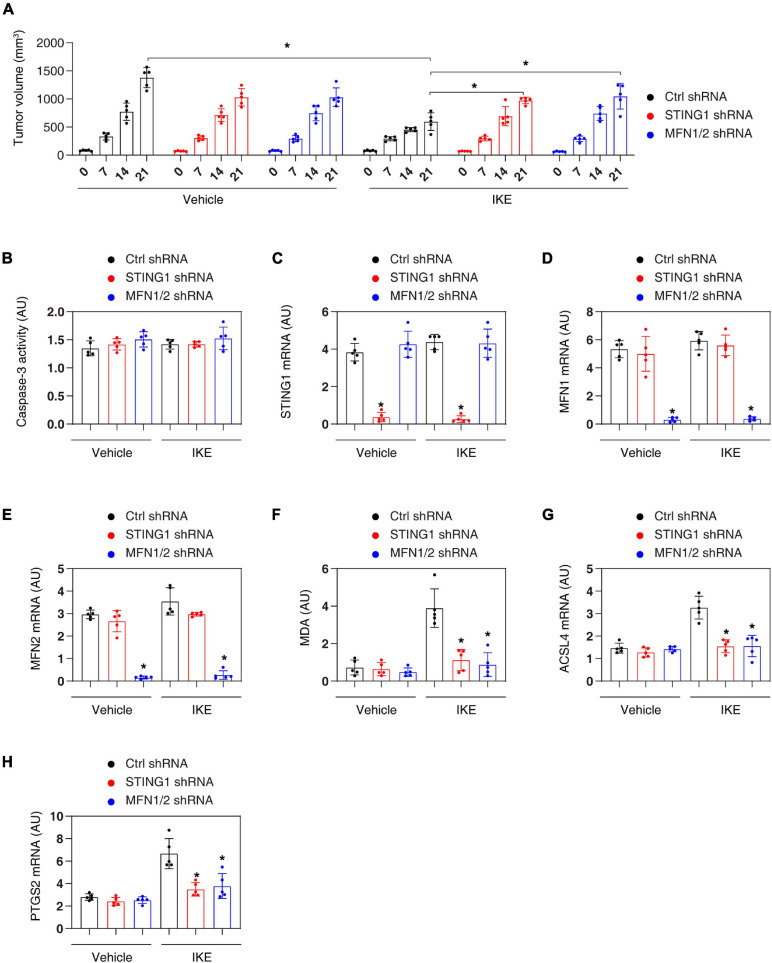
Inhibiting the STING1-MFN1/2 pathway limits ferroptosis therapy *in vivo*. **(A)** NOD SCID mice were injected subcutaneously with the indicated PANC1 cells (5 × 10^6^/mouse) and treated with IKE (40 mg/kg, once every other day by i.p.) at day 7 for 2 weeks. Tumor volume was calculated weekly (*n* = 5 mice/group; ANOVA with Tukey’s multiple comparisons test; *P < 0.05). **(B–H)** In parallel, the activity or levels of caspase-3, indicated gene expression, and MDA in isolated tumors at day 28 were assayed (*n* = 5 mice/group; ANOVA with Tukey’s multiple comparisons test; *P < 0.05 versus control shRNA group). Data are from two independent experiments.

To determine whether the STING1-mitofusion pathway regulates the sensitivity of ferroptosis *in vivo*, we implanted control, STING1^*KD*^, or MFN1/2^*KD*^ PANC1 cell lines into the subcutaneous space on the right flank of nude mice. After 1 week, these mice were administrated imidazole ketone erastin (IKE), a metabolically stable analog of erastin *in vivo* (Zhang et al., 2019). Compared with the control group, IKE-mediated tumor suppression in the STING1^*KD*^ or MFN1/2^*KD*^ group was limited (Figure 5A). The activity of caspase-3 (an apoptosis marker) (Figure 5B) and the mRNA expression of STING1 (Figure 5C), MFN1 (Figure 5D), and MFN2 (Figure 5E) in tumor extracts was not changed by IKE. In contrast, IKE induced the upregulation of malondialdehyde (MDA, one of the products of polyunsaturated fatty acid peroxidation) (Figure 5F). The expression of ferroptosis markers, such as acyl-CoA synthetase long chain family member 4 (ACSL4) (Yuan et al., 2016b; Figure 5G) and prostaglandin-endoperoxide synthase 2 (PTGS2) (Yang et al., 2014; Figure 5H) in tumor extracts was reduced in the STING1^*KD*^ or MFN1/2^*KD*^ group. These animal studies further support the *in vitro* findings that the activation of the STING-MFN1/2 pathway promotes ferroptosis-mediated tumor suppression.

## Discussion

Mitochondria regulate various types of cell death, but the underlying molecular mechanisms for each type are different (Bock and Tait, 2020). In this study, we found that STING1 has a role in promoting ferroptosis by enhancing mitochondrial MFN1/2 function by protein-protein interaction. These findings not only demonstrate a previously unrecognized function of STING1 in modulating mitochondrial dynamics, but also indicate that mitochondrial fusion is important for erastin-induced ferroptosis.

Successful cancer treatment may require the selective killing of cancer cells without affecting normal cells. The term “ferroptosis” was originally used to describe a type of iron-dependent non-apoptotic cell death to selectively remove cancer cells with RAS mutations (Dolma et al., 2003; Dixon et al., 2012). Recent studies have shown that ferroptotic cancer cell death can be triggered in an either RAS-dependent or -independent manner, and many oncogenes and tumor suppressor factors affect the sensitivity of ferroptosis in a context-dependent manner (Xie et al., 2016; Chen et al., 2021a). Consistently, different subcellular organelles (including mitochondria) also play a complex role in regulating ferroptosis in response to different stimuli (Alborzinia et al., 2018; Gao et al., 2018, 2019; Zou et al., 2020). For example, early studies have shown that when using mitochondrial DNA (p0)-depleted cells, mitochondria may not be important for inducing ferroptosis (Dixon et al., 2012). Later, a large number of studies showed that the increase of mtROS (Neitemeier et al., 2017), mitochondrial iron (Yuan et al., 2016a), and tricarboxylic acid cycle metabolites (Gao et al., 2019) selectively contribute to ferroptosis in certain cells. In addition, mitochondrial DNA damage caused by zalcitabine induces ferroptosis by activating the STING1-dependent DNA sensing pathway and subsequent autophagy-dependent cell death (Li C. et al., 2020).

In this study, we determined that STING1 mediates erastin-induced ferroptosis by activating mitochondrial fusion. This different function of STING1 in ferroptosis may depend on its location and stimuli. ER-related STING1 is important for the activation of DNA sensing and autophagy-dependent ferroptosis (Neitemeier et al., 2017; Li C. et al., 2020), while mitochondrial STING1 is the partner of MFN1/2, leading to mitochondrial fusion-dependent ferroptosis. It will be interesting to identify the mitochondrial targeting sequence of STING1 and the structural basis for the binding of STING1 to MFN1/2 during ferroptosis in the future. Our data also indicate that the relationship between mitochondrial STING accumulation and mtROS production is interactive. The increase of mtROS promotes the accumulation of mitochondrial STING, which further amplifies the production of mtROS for ferroptosis.

Autophagy is a degradation mechanism that maintains cell homeostasis in response to various environmental stresses (Levine and Kroemer, 2019), including lipid metabolism dysfunction (Xie et al., 2020). In addition to autophagy’s pro-survival function, excessive autophagy may lead to cell death, which is named “autophagy-dependent cell death” (Galluzzi et al., 2018; Tang et al., 2019). In some cases, ferroptosis appears to be a form of autophagy-dependent cell death (Liu J. et al., 2020). In particular, several types of selective autophagy [e.g., ferritinophagy (Hou et al., 2016), lipophagy (Bai et al., 2019), and clockophagy (Yang et al., 2019)] promote ferroptosis by degrading anti-ferroptosis regulators. In contrast, the function of mitophagy in ferroptosis is still uncertain and even contradictory. For example, CCCP-induced mitophagy inhibits cysteine deprivation- or erastin-induced ferroptosis in HT1080 cells with exogenously expressed PRKN (Gao et al., 2019), although other studies suggested that mitophagy has no effect on ferroptosis caused by erastin or RSL3 in same PRKN-expressed HT1080 cells (Gaschler et al., 2018). In the current study, we found that the depletion of PINK1 in PANC1 cells failed to affect erastin-induced ferroptotic cell death. It remains to be seen whether PINK1-independent mitophagy pathways (e.g., BCL2 interacting protein 3-like [BNIP3L, also known as NIX]; Sandoval et al., 2008) regulate erastin-induced ferroptosis, although the total level of mitochondria was not different in the control group and STING1^*KD*^ cells.

Mitochondrial dynamics are a feature of the interaction between mitochondria and other organelles. In general, mitochondrial fragmentation caused by excessive mitochondrial fission is a common event in apoptotic cells, which may disrupt the oxidative phosphorylation (OXPHOS) process (Youle and Karbowski, 2005). Mitochondrial fusion is important to maintain structural and genetic mitochondrial integrity (Vidoni et al., 2013). In particular, erastin increases mitochondrial fusion, which may generate mtROS during OXPHOS or increase the conversion of fatty acids to acetyl-CoA through mitochondrial fatty acid β-oxidation (Liesa and Shirihai, 2013). Finally, the increased supply of ROS and fatty acids during mitochondrial fusion can lead to lipid peroxidation, leading to ferroptosis. These findings indicate that the dynamic state of mitochondria may determine the selectivity of apoptosis and ferroptosis.

In summary, we reported a novel role of STING1 in promoting MFN1/2-dependent mitochondrial fusion and subsequent ferroptosis. Targeting mitochondrial fusion-mediated ferroptotic cell death may represent a promising cancer treatment strategy in the future. Nevertheless, a more comprehensive approach is essential to enhance the pro-ferroptotic effect of STING1 without changing its beneficial mechanism in tumor immunity. Since STING1 plays a critical role in innate immunity, it is still interesting whether mitochondrial STING1-mediated ferroptosis is implicated in infection and sterile injury (Chen et al., 2021b).

## Materials and Methods

### Reagents

Zalcitabine (S1719), erastin (S7242), sulfasalazine (S1576), IKE (S8877), MitoQ (S8978), bafilomycin A1 (S1413), staurosporine (S1421), and CCCP (S6494) were purchased from Selleck Chemicals. MitoTEMPO (SML0737) was purchased from Sigma-Aldrich. The antibodies to STING1 (13647), TOMM20 (42406), MFN1 (14739), OPA1 (67589), PINK1 (6946), PRKN (4211), ACTB (3700), CALR (12238), and MAP1LC3B (3868) were purchased from Cell Signaling Technology. The antibody to MFN2 (ab205236) was purchased from Abcam. The antibody to mitHsp70 (MA3-028) was purchased from Thermo Fisher Scientific.

### Cell Culture

The PANC1 (CRL-1469), Capan2 (HTB-80), and MFN1/2^–/–^ MEFs (CRL-2994) were purchased from the American Type Culture Collection. These cell lines were grown at 37°C, 95% humidity, and 5% CO_2_ according to standard mammalian tissue culture protocols and aseptic techniques. In brief, PANC1 and MEFs were cultured in Dulbecco’s Modified Eagle’s Medium (Sigma-Aldrich, D6429), whereas Capan2 was cultured in McCoy’s 5a Medium Modified (Sigma-Aldrich, M4892). All media were supplemented with 10% heat-inactivated (56°C for 30 min) fetal bovine serum (Sigma-Aldrich, F4135) and 5000 units/ml of penicillin and 5000 μg/ml of streptomycin (Thermo Fisher Scientific, 15070063). The identity of the cell line was verified by short tandem repeat analysis, and routine mycoplasma testing was negative for contamination.

### Animal Study

A PANC1-derived xenograft tumor model was established as previously described. In short, wild-type, STING1^*KD*^ or MFN1/2^*KD*^ PANC1 cells (5 × 10^6^ cells) were injected subcutaneously into the dorsal side of NOD SCID mice (female, 8–10 weeks old). On the 7th day, these mice were given IKE (40 mg/kg, once every other day, intraperitoneal injection) for 2 weeks. The diameter of the tumor was measured twice a week with a caliper, and the tumor volume was calculated using the following formula: length × width^2^ × π/6. At 14 days after the injection, the mice were euthanized, and the xenograft solid tumors were collected. All mice were maintained under specific pathogen-free conditions. All animal experiments were conducted in accordance with the institutional ethical guidelines related to animal care and were approved by the Institutional Animal Health and Use Committee.

### Cell Death Assay

A Countess II FL Automatic Cell Counter (Thermo Fisher Scientific, AMQAF1000) was used to determine the percentage of dead cells after staining with 0.4% trypan blue solution (Sigma-Aldrich, T8154). Trypan blue is a negatively charged dye that can only stain cells with damaged cell membranes, thereby indicating cell death.

### RNAi

The following human shRNAs were obtained from Sigma-Aldrich:*STING1* shRNA (sequence: CCGGGCTGGCATGGTCA TATTACATCTCGAGATGTAATATGACCATGCCAGCTTTTT TG), *STING1* shRNA-2 (sequence: CCGGGCTGTATA TTCTCCTCCCATTCTCGAGAATGGGAGGAGAATATACAG CTTTTTTG), *MFN1* shRNA (sequence: CCGGGCTCCCATT ATGATTCCAATACTCGAGTATTGGAATCATAATGGGAGC TTTTTG), *MFN2* shRNA (sequence: CCGGGTCAAAGG TTACCTATCCAAACTCGAGTTTGGATAGGTAACCTTTGAC TTTTTG), *PINK1* shRNA (sequence: CCGGCGGACG CTGTTCCTCGTTATGCTCGAGCATAACGAGGAACAGCGT CCGTTTTTTG), and *PRKN* shRNA (sequence: CCGGGGCCT ACAGAGTCGATGAAAGCTCGAGCTTTCATCGACTCTGTA GGCCTTTTTG). Lipofectamine 3000 (Thermo Fisher Scientific, L3000001) was used for shRNA transfection according to manufacturer’s guidelines. In short, when the cells reached 70–80% confluence at the time of transfection, the DNA-lipid complex was added to the cells using serum-free Opti-MEM medium. After 48–72 h of transfection, the efficiency of RNAi was detected by qRT-PCR or western blotting.

### qRT-PCR Assay

According to the manufacturer’s instructions, we used an QIAGEN RNeasy Plus Micro Kit (74034) and iScript cDNA Synthesis Kit (Bio-Rad, 1708890) to extract total RNA and synthesize first-strand cDNA, respectively. We performed qRT-PCR using synthetic cDNA and primers (*STING1*: 5′-CCTGAGTCTCAGAA CAACTGCC-3′ and 5′-GGTCTTCAAGCTGCCCACAGTA-3′; *PTGS2*: 5′-CGGTGAAACTCTGGCTAGACAG-3′ and 5′-GCAAACCGTAGATGCTCAGGGA-3′; *ACSL4*: 5′-GCTATCT CCTCAGACACACCGA-3′ and 5′-AGGTGCTCCAACTCTGC CAGTA-3′; *PINK1*: 5′-GTGGACCATCTGGTTCAACAGG-3′ and 5′-GCAGCCAAAATCTGCGATCACC-3′; and *PRKN*: 5′-CCAGAGGAAAGTCACCTGCGAA-3′ and 5′-CTGAGGCTTC AAATACGGCACTG-3′). The data were normalized to *RNA18S* (5′-CTACCACATCCAAGGAAGCA-3′ and 5′-TTTTTCGTCACTACCTCCCCG-3′). Based on the untreated group, the relative concentration of mRNA was expressed in arbitrary units, and its assigned value was 1.

### Detection of Mitochondrial DNA Damage

Mitochondrial DNA damage was assayed using a kit from Thermo Fisher Scientific (50-753-4394) according to the manufacturer’s instructions. This DNA damage detection kit was used to determine the damaged 8.1–8.8 kb mitochondrial DNA sequences by quantification of the replicated DNA with real-time PCR following qPCR analysis.

### Western Blot Analysis

After treatment, whole cells or organelle fractions were harvested and lysed at 4°C in ice-cold Cell Lysis Buffer (Cell Signaling Technology, 9803) containing a protease inhibitor cocktail (Sigma-Aldrich, P0044) (Tang et al., 2010). A bicinchoninic acid (BCA) assay was used to detect protein concentration, and then 30 μg of protein in each lysate sample was subjected to SDS-PAGE (Bio-Rad, 3450124) at 100–120 V for 90 min, and then transferred to PVDF membrane (Bio-Rad, 1704273). The PVDF membrane was blocked with 5% non-fat dry milk, and then incubated with the indicated primary antibody (1:500-1:1000) at 4°C overnight. The membrane was washed three times in TBS-T buffer, and then incubated with an HRP-linked secondary antibody for 1 h at room temperature. After enhanced chemiluminescence exposure, a ChemiDoc Touch Imaging System (Bio-Rad, 1708370) was used for visualization and quantitative analysis of protein expression according to the manufacturer’s instructions. ACTB was used as a housekeeping control for whole cell lysates. TOMM20 and CALR (also known as calreticulin) were used as a mitochondrial or ER loading control, respectively. Mitochondria and ER isolation kits were obtained from Thermo Fisher Scientific (89874) or Sigma-Aldrich (ER0100), respectively.

### Immunoprecipitation Analysis

After treatment, the cells were lysed at 4°C using ice-cold RIPA lysis buffer (Cell Signaling Technology, 9806), and the cell insoluble matter was removed by centrifugation (12,000 *g*, 10 min). BCA was used to detect the protein concentration, and then 200-300 μg protein in each lysate sample was pre-cleared for 3 h with Protein A Sepharose beads (Cell Signaling Technology, 9863) at 4°C. Then, in the presence of Protein A Sepharose beads, the sample with control IgG or a specific antibody (5 μg/mL) was gently shaken overnight at 4°C. After incubation, the Protein A Sepharose beads were washed thoroughly with PBS, and the protein was eluted by boiling in 2 × Laemmli sample buffer (Bio-Rad, 161-0737) before SDS-PAGE.

### Biochemical Assay

Commercially available assay kits were used to measure the concentrations or activity or release of MDA (Abcam, ab118970), HMGB1 (Shino-Test Corporation, 326070442), caspase-3 (Sigma-Aldrich, CASP3C), or glutamate (Thermo Fisher Scientific, A12221) in the indicated samples according to manufacturer’s guidelines.

### Probe and Image Assay

Commercially available probes, including lipid peroxidation probe BODIPY-C11 (Thermo Fisher Scientific, D3861), mtROS probe MitoSOX (Thermo Fisher Scientific, M36008), and mitophagy probe Mtphagy Dye (Dojindo, MD01-10) were used in live cells according to manufacturer’s guidelines. Hoechst 33258 dye (Thermo Fisher Scientific, H3569) was used to stain cell nuclei. The signal was analyzed using a confocal microscope (ZEISS) or microplate reader (Tecan). The morphology of mitochondria was determined by staining TOMM20 and then a quantitative assay for mitochondrial fusion was performed as previously described (Bahat et al., 2018).

### Statistical Analysis

Data are expressed as mean ± SD unless otherwise stated. Unpaired Student’s *t*-test or an analysis of variance (ANOVA) were used to compare two or different groups, respectively. A *P*-value of < 0.05 was considered statistically significant.

## Data Availability Statement

The original contributions presented in the study are included in the article/Supplementary Material, further inquiries can be directed to the corresponding author/s.

## Ethics Statement

The animal study was reviewed and approved by the UT Southwestern Medical Center and Jilin University.

## Author Contributions

CL and DT conceived and designed the experiments and wrote the manuscript. CL, JL, WH, RK, and DT performed the experiments. All authors contributed to the article and approved the submitted version.

## Conflict of Interest

The authors declare that the research was conducted in the absence of any commercial or financial relationships that could be construed as a potential conflict of interest. The handling editor declared a shared affiliation, though no other collaboration, with one of the author WH at the time of the review.
