# Supplementary material for: STING1 Promotes Ferroptosis Through MFN1/2-Dependent Mitochondrial Fusion
Source: Front Cell Dev Biol. 2021 Jun 14;9:698679. doi: 10.3389/fcell.2021.698679 (PMC8236825; doi:10.3389/fcell.2021.698679)
Supplement: Supplementary file 1 [file Data_Sheet_1.PDF]

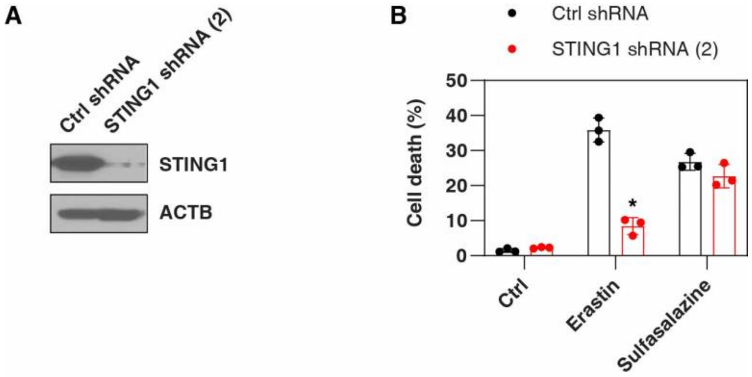

Figure S1. STING1 promotes erastin-induced ferroptosis. (A) Western blot analysis of STING1 expression in control and $STING^{KD}$ PANC1 cells. (B) Indicated PANC1 cells were treated with erastin (10 μM) or sulfasalazine (500 μM) for 24 hours, and then cell death was assayed (n = 3 wells/group; ANOVA with Tukey's multiple comparisons test; *P < 0.05 versus control shRNA group).
